# An Adaptive Signal Denoising Method Based on Reweighted SVD for the Fault Diagnosis of Rolling Bearings

**DOI:** 10.3390/s25082470

**Published:** 2025-04-14

**Authors:** Baoxiang Wang, Chuancang Ding

**Affiliations:** 1 School of Mechanical Engineering, Suzhou University of Science and Technology, Suzhou 215009, China; wangbaoxiang@usts.edu.cn; 2 School of Rail Transportation, Intelligent Urban Rail Engineering Research Center of Jiangsu Province, Soochow University, Suzhou 215131, China

**Keywords:** singular value decomposition, frequency domain multipoint kurtosis, signal denoising, rolling element bearings, fault diagnosis

## Abstract

**Highlights:**

**What are the main findings?**

- This paper proposed an adaptive signal denoising method based on frequency domain multipoint kurtosis (FDMK) and singular value decomposition (SVD).
- FDMK is used to identify sensitive singular components that contain fault-related information, and an estimation process is proposed to calculate the fault characteristic frequency.

**What is the implication of the main finding?**

- The proposed method FDMK-SVD can effectively extract fault features from raw vibration signals, even when faced with significant background noise and other interferences, enabling accurate fault diagnosis of rolling bearings.

**Abstract:**

Due to the harsh and complex operating conditions, rolling element bearings (REBs) are prone to failures, which can result in significant economic losses and catastrophic breakdowns. To efficiently extract weak fault features from raw signals, singular value decomposition (SVD)-based signal denoising methods have been widely adopted in the field of rolling bearing fault diagnosis. In traditional SVD-based methods, singular components (SCs) with significant singular values are selected to reconstruct the denoized signal. However, this approach often overlooks low-energy SCs that contain important fault information, leading to inaccurate diagnosis. To address this issue, we propose a new selection scheme based on frequency domain multipoint kurtosis (FDMK), along with a reweighting strategy based on FDMK to further emphasize weak fault features. In addition, the estimation process of fault characteristic frequency is introduced, allowing FDMK to be calculated without prior information. The proposed FDMK-SVD can adaptively extract periodic fault features and accurately identify the health condition of REBs. The effectiveness of FDMK-SVD is validated using both simulated and experimental data obtained from a locomotive bearing test rig. The results show that FDMK-SVD can effectively extract fault features from raw vibration signals, even in the presence of severe background noise and other types of interferences.

## 1. Introduction

Rolling element bearings (REBs) are essential components of rotating machinery and play a significant role in the modern industry. Due to their harsh operating conditions, REBs are prone to unexpected failures, which can lead to machine breakdowns and substantial economic losses [1,2]. Consequently, the condition monitoring of REBs has become crucial, attracting significant attention from both academia and industry [3,4].

Since the vibration signal contains abundant fault information that can effectively reflect the health conditions of REBs, vibration analysis-based diagnostic methods have been widely applied for fault diagnosis [5]. When a fault occurs in a bearing, a series of repetitive impulses with a unique period are produced in the vibration signal and excite the resonances of the bearing and mechanical system [6,7]. Therefore, fault diagnosis of REBs can be achieved by detecting the periodical fault impulses in the time domain waveform or fault characteristic frequency (FCF) in the envelope spectrum domain. However, fault-induced repetitive transients are often submerged by strong background noise and other interferences, making fault diagnosis in real-world applications challenging. Therefore, extracting the repetitive impulses from the raw vibration signal is a critical and difficult task in the fault detection of REBs.

In recent years, many researchers have worked to address this issue, proposing various diagnostic methods, including spectral kurtosis [8,9], wavelet transform (WT) [10], empirical mode decomposition (EMD) [11,12], minimum entropy deconvolution (MED) [13,14,15], and singular value decomposition (SVD). Among these methods, SVD has garnered significant attention due to its ability to perform adaptive decomposition and its high computational efficiency.

Due to the abovementioned merits, SVD-based signal denoising methods have been widely employed in condition-based maintenance (CBM). Zhao et al. [16] illustrated the similarities between SVD and WT when the Hankel matrix is selected to convert one-dimension discrete signal into two-dimension matrix. On the basis of the above findings, a singular value decomposition packet (SVDP) algorithm was developed for decomposing vibration signals and extracting weak fault features [17]. Moreover, SVD has been employed to enhance the signal-to-noise ratio (SNR) in both the time and frequency domains [18], which efficiently promotes the improvement of the reliability of fault detection. Cong et al. presented a hybrid method consisting of SVD, singular value ratio, and short-time matrix series for the vibration signal analysis [19], which achieves an accurate diagnosis of rolling bearings. Duan et al. [20] proposed an impulsive feature extraction with improved SVD and sparsity-closing morphological analysis, in which A length factor is designed to optimize the construction of the matrix. Jiang et al. [21] combined SVD with the whale optimization algorithm for the quantitative detection of multiple damages in turbine blades. In addition to the SVD-based signal denoising method, SVD can also be combined with other fault diagnosis methods, where SVD is utilized to extract the fault feature for further signal processing. Jiang et al. [22] proposed a novel method combining SVD with a continuous hidden Markov model, achieving high classification accuracy in rolling bearing diagnosis. In addition, Kang et al. [23] presented an SVD-based method for fault classification, in which the features extracted using SVD were adopted as the input of the multi-layer support vector machines. Muruganatham et al. [24] took the singular value or the energy of SCs as the input features for the neural network and successfully achieved the automated fault diagnosis of REBs.

The key to the SVD-based signal denoising method is to determine an appropriate criterion for selecting the SC for subsequent signal reconstruction. A difference spectrum [25] was proposed to capture the sudden change point, which reflects the boundary between the signal and noise. These change points are then used to select effective singular values. A similar approach based on the correlation coefficient was introduced in [26]. However, periodicity and impulsiveness are not taken into account in the above two methods, which may cause effective singular values to be ignored and even incorrect diagnostic results. To address this issue, a novel information index, the periodic modulation intensity (PMI) [27], was used to evaluate whether the SCs contained diagnostic information. This index helps to accurately select SCs that carry fault-related information, thereby improving the reliability of fault diagnosis. However, one challenge of PMI is that the fault period of interest must be predetermined, which can hinder its application in real-world situations in which prior knowledge of the fault period is not always available.

To overcome the drawbacks of the abovementioned SVD-based signal denoising method, this paper proposes a new selection scheme based on frequency domain multipoint kurtosis (FDMK) to select the effective SCs. FDMK evaluates SCs based on their periodicity and impulsiveness, ensuring the accurate selection of SCs that contain fault-related information, unlike traditional SVD-based methods that retain only large-energy SCs. Additionally, a reweighting strategy based on FDMK is introduced to enhance weak fault features during the reconstruction of the denoized signal. In addition, to solve the problem of the fault characteristic frequency or fault period not being given in advance, the process of estimating the fault period was incorporated, which allowed the proposed FDMK-SVD to operate effectively without prior knowledge of the fault period or characteristic frequency. Therefore, the proposed FDMK-SVD can adaptively achieve fault diagnosis and health assessment of REBs without prior information.

The remainder of this paper is organized as follows. In Section 2, the principle of the SVD-based signal denoising method is briefly introduced. In Section 3, the proposed FDMK-SVD for fault diagnosis of REBs is presented. Specifically, Section 3.1 and Section 3.2 introduce the definition of FDMK and the estimation process of fault characteristic frequency (FCF), respectively, and the detailed flowchart of the FDMK-SVD is illustrated in Section 3.3. Section 4 presents the preliminary validation of the FDMK-SVD using a simulated signal. Subsequently, in Section 5, the vibration signal acquired from the experiment is used to further confirm the effectiveness of FDMK-SVD. Finally, the conclusions are summarized in Section 6.

## 2. Principle of SVD-Based Signal Denoising Approach

For a real matrix ***A***∈*R^m×n^*, the SVD of the matrix ***A*** can be defined as follows [16,25].

(1) $$A=U\Sigma V^{T}$$

where the matrices ***U***= [*u*_1_, *u*_2_, …, *u_m_*]∈*R^m^*^×*m*^ and matrix ***V*** = [*v*_1_, *v*_2_, …, *v_n_*]∈*R^n^*^×*n*^ are orthogonal to each other. The column vectors of matrices ***U*** and ***V*** are the eigenvectors of ***AA****^T^* and ***A****^T^**A***, respectively. **Σ** = [diag(*σ*_1_, *σ*_2_, …, *σ*_k_), **0**]∈*R*^m×n^ is a diagonal matrix, where **0** is a zero matrix, *k* = min(*m*, *n*), *σ* is the singular value of matrix ***A***, and *σ*_1_ ≥ *σ*_2_, …, ≥ *σ*_k_.

As illustrated in [25,27,28], SVD is effectively used for vibration signal denoising, which can be achieved through the following steps:

Step 1: Construction of Hankel matrix

In practice, the vibration signal is usually a one-dimensional time series rather than a matrix; thus, converting the one-dimensional signal into a matrix ***A*** is indispensable. Some matrix forms have been presented, such as the Toeplitz matrix, the cycle matrix, and the Hankel matrix. Because the Hankel matrix has zero phase-shifting characteristics and wavelet-like properties, the Hankel matrix is employed in [16].

The Hankel matrix of the discrete signal ***x*** = [***x***(1), ***x***(2), …, ***x***(*N*)] can be written as [27]

(2) $$A=\left[\begin{array}{cccc} x(1) & x(2) & \cdots & x(n)\\ x(2) & x(3) & \cdots & x(n+1)\\ \vdots & \vdots & \ddots & \vdots \\ x(m) & x(m+1) & \cdots & x(N) \end{array}\right]$$

where 1 < *n* < *N* and *m* + *n* − 1 = *N*. Parameter *m* is usually selected less than *n*, and it is suggested to set as 3 times the number of sub-components of the vibration signal [29]. This choice of *m* also determines the number of SCs after performing SVD.

Step 2: Signal decomposition and reconstruction

Once matrix ***A*** is constructed, it can be expressed as the product of matrices ***U***, **∑,** and ***V****^T^* according to Equation (1). This means matrix ***A*** can be decomposed into a sum of unit-rank elementary matrices that are mutually orthogonal, which can be expressed as

(3) $$\begin{array}{c} A=\left[u_{1},u_{2},\ldots ,u_{m}\right]\left[\begin{array}{ccccc} \sigma_{1} & 0 & \cdots & 0 & 0\\ 0 & \sigma_{2} & \cdots & 0 & 0\\ \vdots & \vdots & \ddots & 0 & 0\\ 0 & 0 & \cdots & \sigma_{m} & 0 \end{array}\right]\left[\begin{array}{c} v_{1}^{\tau }\\ v_{2}^{\tau }\\ \vdots \\ v_{n}^{\tau } \end{array}\right]\\ =\sigma_{1}u_{1}v_{1}^{\tau }+\sigma_{2}u_{2}v_{2}^{\tau }+\ldots +\sigma_{m}u_{m}v_{m}^{\tau }\\ =A_{1}+A_{2}+\ldots +A_{m} \end{array}$$

where ***u***_i_∈***R****^m^*^×1^ and ***v***_i_∈**R***^m^*^×1^ are the *i*th column vectors of matrices ***U*** and ***V***, respectively.

**Figure 1:**
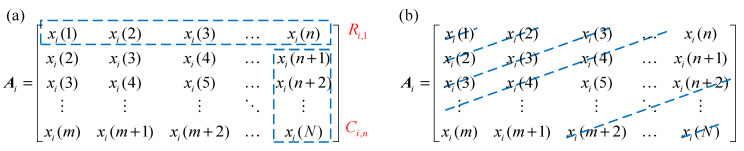
Obtaining ***x****_i_* from the sub-matrix ***A****_i_*. (**a**) direct method; and (**b**) anti-diagonals averaging method.

As illustrated in Equation (3), matrix ***A*** can be decomposed into the sum of sub-matrix ***A****_i_*, and each singular component ***x****_i_* corresponding to sub-matrix ***A****_i_* can be obtained using two methods, as demonstrated in Figure 1. In the direct method, the singular component ***x****_i_* can be obtained by concatenating the first row and sub-column vector in the last column of ***A****_i_*, which can be written as

(4) $$x_{i}=\left[R_{i,1},C_{i,n}^{T}\right]$$

where ***R****_i_*_,1_∈***R****^n^*^×1^ and ***C****_i_*_,*n*_∈***R***^(*m*−1)×1^. Another way to obtain the singular component *x*_i_ is by diagonal averaging, where each element of ***x***_i_ can be obtained by averaging along the anti-diagonals, as shown in Figure 1b. Interestingly, regardless of any of the methods mentioned above being used, the raw signal can be perfectly reconstructed by adding all decomposed SCs, as shown in Equation (5).

(5) $$x=\sum_{i=1}^{m}x_{i}$$

Step 3: Signal denoising

As illustrated in Equations (3) and (5), each singular value is associated with an SC. This means that the selection of effective SCs can be transformed into the selection of the corresponding singular values. Therefore, the SVD-based signal denoising method can be achieved by selecting some effective SCs and simply adding their corresponding component signals together. In general, the SCs with large singular values are considered as the effective components and would be retained [25], which can be expressed as

(6) $$\tilde{A}=\left[u_{1},u_{2},\ldots ,u_{m}\right]\left[\begin{array}{ccccc} \sigma_{1} & 0 & \cdots & 0 & 0\\ 0 & \sigma_{2} & \cdots & 0 & 0\\ \vdots & \vdots & \ddots & \vdots & \vdots \\ 0 & 0 & \cdots & \sigma_{k} & 0\\ 0 & 0 & \cdots & 0 & 0 \end{array}\right]\left[\begin{array}{c} v_{1}^{\tau }\\ v_{2}^{\tau }\\ \vdots \\ v_{n}^{\tau } \end{array}\right]=\sum_{i=1}^{k}\sigma_{i}u_{i}v_{i}^{\tau }=\sum_{i=1}^{k}A_{i}\Rightarrow \tilde{x}=\sum_{i=1}^{k}x_{i}$$

where the first *k* largest singular values are added together; $\tilde{A}$ and $\tilde{x}$ represent the denoized matrix and signal, respectively.

In the abovementioned SVD-based signal processing approach, it is important to determine the proper threshold *k* to guarantee denoising performance. The selection of *k* has been investigated in several studies, such as the difference spectrum [25], correlation coefficients [26], and the median value of singular values [28]. Among these methods, the difference spectrum is frequently adopted because of its easy implementation and good performance in applications. More details about the difference spectrum can be found in [28].

## 3. Reweighted Singular Value Decomposition Based on Frequency Domain Multipoint Kurtosis

### 3.1. Frequency Domain Multipoint Kurtosis

In the fault diagnosis of REBs, many indicators can be used to detect whether the signal contains fault information and quantify the extent of this information. Common indicators include Shannon entropy [30,31], Gini index [32], kurtosis [33], and *l*_2_/*l*_1_ norm [34]. However, most of these indicators mentioned above are introduced from the field of statistics, economics, and information theory, and the periodicity of the defect impulses caused by defective rotating machinery is not considered. In order to take into account the periodicity of the defect impulses, several other indicators, such as correlated kurtosis [35], envelope harmonic-to-noise ratio (EHNR) [36], and multipoint kurtosis (MK) [37], have been proposed in the literature. In this paper, the multipoint kurtosis that considers the periodicity of the defect impulses is utilized to select the sensitive SCs containing the information related to the health conditions of REBs.

The multipoint kurtosis of *M*-shift of zero-mean data ***y*** is expressed as [37]

(7) $$MK(\vec{y},\vec{t})=\frac{{\left(\sum_{n=1}^{N}t_{n}^{2}\right)}^{2}}{\sum_{n=1}^{N}t_{n}^{8}}\frac{\sum_{n=1}^{N}{\left(t_{n}y_{n}\right)}^{4}}{{\left(\sum_{n=1}^{N}y_{n}^{2}\right)}^{2}}$$

where *N* is the length of the original signal ***y*** and $\vec{t}$ is the target vector. For example, $\vec{t}$ can be defined as

(8) $$\vec{t}={\left[\begin{array}{cccccccccccc} 0 & 0 & 0 & 1 & 0 & 0 & 0 & 1 & 0 & \ldots & 0 & 1 \end{array}\right]}^{T}$$

The above $\vec{t}$ controls the locations of the interested multiple points and the interval between adjacent peaks or impulses, where the interval is four points and the first impulse is located at the 4th point.

Although the MK can extract repetitive impulses, a major drawback still exists that hinders its application. As demonstrated in Equation (8), the locations of the impulses are assumed to be known in advance. However, it is impossible to determine the locations of fault impulses in actual situations, which makes it unrealistic to calculate the MK directly in the time domain. As illustrated in the previous literature, the position of the frequency lines associated with the FCF and its harmonics is constant in the envelope spectrum for periodic fault transients, regardless of their positions in the time waveform. Therefore, it is practical to calculate the MK in the frequency domain (envelope spectrum), and FDMK can be defined by

(9) $$FDMK(\vec{y},\vec{t})=\frac{{\left(\sum_{n=1}^{N}t_{n}^{2}\right)}^{2}}{\sum_{n=1}^{N}t_{n}^{8}}\frac{\sum_{n=1}^{N}{\left(t_{n}S_{n}\right)}^{4}}{{\left(\sum_{n=1}^{N}S_{n}^{2}\right)}^{2}}$$

where ***S*** represents the envelope spectrum of the signal and $\vec{t}$ represents the target vector. According to this definition, it can be observed that the FCF and sampling frequency *f_s_* must be predetermined to construct the target vector $\vec{t}$. However, it is sometimes impossible to obtain the FCF in advance, or the theoretical FCF deviates from the real value, which leads to an inaccurate FDMK and even incorrect diagnostic results. To address this issue, a new strategy that can adaptively estimate the fault characteristic frequency is presented in the next section.

### 3.2. The Estimation of Fault Characteristic Frequency and Construction of Target Vector $\vec{t}$

As demonstrated in [38,39], the autocorrelation function can be utilized to estimate the period of the fault impulses. The definition of autocorrelation function of ***x***(*t*) is expressed as follows

(10) $$r_{x}(\tau )=\int x(t)x(t+\tau )dt$$

**Figure 2:**
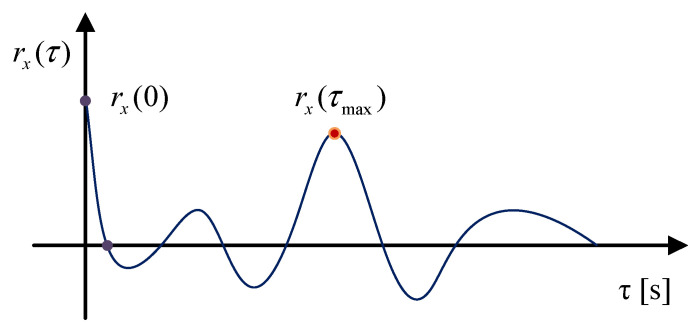
The autocorrelation function of *x*(*t*).

As illustrated in Figure 2, *τ* is the time lag, and the autocorrelation function of ***x***(*t*) reaches a local maximum when *τ* = *τ*_max_. Therefore, *τ*_max_ is considered the fault period.

However, the above method for estimating the period or FCF through the envelope autocorrelation function of the raw vibration signal may fail when the defect impulses are submerged by harmonics or severe background noise. To overcome these limitations, a new strategy based on SVD to estimate the period or fault characteristic frequency is proposed in this paper.

Step 1: Obtain the singular components using SVD.

In real applications, defect impulses are usually overwhelmed by harmonic components and background noise, leading to inaccurate estimates of the fault period or FCF from the raw signal. Therefore, SVD is adopted to separate the components in the raw signal, and SCs are obtained.

Step 2: Calculate the Gini index (GI) of each SC and then calculate the envelope harmonic-to-noise ratio (EHNR) to select the most sensitive SC.

In this part, GI and EHNR are introduced to select the most sensitive SC containing the diagnostic information. The definitions of GI and EHNR are provided below.

The Gini index (GI) was originally used to represent the wealth distribution of a nation’s population. In recent years, the GI has been increasingly used as a measure of signal sparsity [32,40,41]. The GI values range from 0 to 1, and a large GI value indicates that the signal is sparser. For a real signal indexed in non-decreasing order, its GI can be given below

(11) $$GI(x)=1-2\sum_{i=1}^{N}\frac{\left|x_{i}\right|}{{\left\|x\right\|}_{1}}\left(\frac{N-i+0.5}{N}\right)$$

where ||***x***||_1_ represents the *l*_1_-norm of ***x*** and *N* is its length.

EHNR is proposed for detecting periodic impulses [39,42], and the formula is defined as follows:

(12) $$EHNR=\frac{r_{E[x(t)]}\left(\tau_{\max}\right)}{r_{E[x(t)]}\left(0\right)-r_{E[x(t)]}\left(\tau_{\max}\right)}$$

where *E*[***x***(*t*)] represents the envelope of signal***x***, and the autocorrelation function of *E*[***x***(*t*)] reaches a local maximum when *τ* = *τ*_max_.

After separating the components contained in the raw signal, the GI value of each singular component is calculated, and the EHNR of the first several SCs with large GI values are further calculated. In this way, the periodicity and sparsity of the defect impulse are considered at the same time. The SC with the maximum ENHR is considered the most sensitive one, which is related to the fault feature.

Step 3: Estimate the fault period or fault characteristic frequency

The envelope autocorrelation function of the selected SC is calculated, and the time lag *τ*_max_ that causes the envelope autocorrelation function to reach a local maximum is considered the fault period. Correspondingly, the fault characteristic frequency is 1/*τ*_max_.

### 3.3. The Proposed FDMK-SVD for Fault Detection of REBs

**Figure 3:**
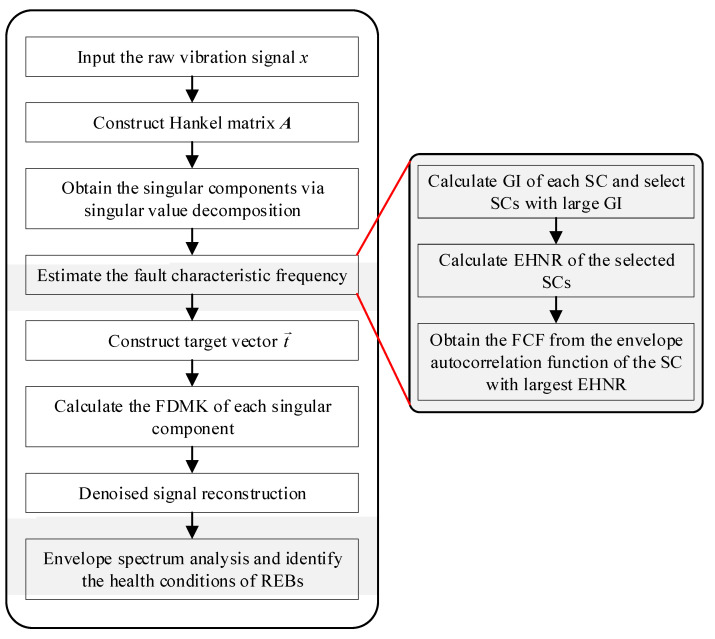
The flowchart of the proposed FDMK-SVD.

The framework of the proposed FDMK-SVD for bearing fault detection is shown in Figure 3, and the concrete steps are presented as follows:

Step 1: Signal decomposition

A one-dimensional signal must first be converted into a Hankel matrix, as illustrated in Section 2 for SVD. When a relatively large *m* is selected, the raw signal, consisting of fault impulse components, harmonic components, and other interferences, can be separated, and the computational cost increases as *m* increases. According to the analysis of the simulated signal and experimental signals, *m* = 30 is appropriate for separating the components contained in the vibration signal under different situations.

Step 2: Estimate the fault characteristic frequency

After obtaining SCs, GI and EHNR are set as the criteria to select the most sensitive SC, followed by the estimation of the fault period or FCF using the envelope autocorrelation function of the selected SC.

Step 3: Construct the target vector $\vec{t}$After obtaining the characteristic frequency, the corresponding target vector $\vec{t}$ can be generated. Because the estimated fault characteristic has a slight deviation from the real value, the locations of ‘1’ in the target vector $\vec{t}$ are determined by seeking the locations of the local maximum around the estimated value, which indicates that ‘1’ is not equally distributed.

Step 4: Calculate the FDMK of each singular component

In this step, the FDMK of each singular component is calculated to evaluate whether the diagnostic information is included.

Step 5: Denoized signal reconstruction

The denoized signal is obtained by assigning a new weighting coefficient to each SC and then adding the weighted SCs, as given in Equations (13) and (14).

(13) $$\tilde{X}=w(1)X_{1}+w(2)X_{2}+w(3)X_{3}+\cdots +w(m)X_{m}$$

(14) $$w(i)=\left\{\begin{array}{c} 0\;mk_{i}\leq threshold\\ mk_{i}/\sum_{i}mk_{i}\;mk_{i}>threshold \end{array}\right.$$

In Equation (14), the weighting function ***w***(*i*) determines whether each SC is highlighted or suppressed when reconstructing the denoized signal. The greater the weight value of each SC, the greater its contribution to reconstructing the denoized signal. For each SC, if its corresponding *mk_i_* ≤ *threshold*, then the SC is considered to contain no fault feature information, and it is culled by assigning it a weight of zero when reconstructing the denoized signal. Conversely, if the SCs with *mk_i_* > *threshold*, then it is considered to contain fault information and should be preserved. Furthermore, since different SC contain different levels of information, SC is reweighted by the normalized FDMK, as shown in Equation (14). This ensures that SCs with larger FDMK can be highlighted more effectively in the reconstruction of the denoized signal. In this paper, the threshold is empirically set to 0.02.

Step 6: Envelope spectrum analysis and condition identification

Finally, the envelope spectrum of the reconstructed denoized signal is analyzed to detect the fault characteristic frequencies. By identifying the presence of these frequencies in the envelope spectrum, the health condition of the REBs can be accurately assessed.

## 4. Simulation Analysis

In this section, a simulated signal of a faulty rolling bearing is constructed to verify the effectiveness of the proposed FDMK-SVD. As mentioned above, the bearing vibration signal collected from an actual industrial environment contains multiple signal components while being submerged in heavy background noise. To create a more realistic signal model, we incorporate four common signal components into the simulated signal of a faulty rolling bearing, as described below [43].

(15) $$\begin{array}{c} y(t)=\underset{Rotorvibration}{\underset{︸}{\sum_{n}A_{n}\cos(2\pi nf_{0}t+a_{n})}}+\underset{Gearmeshing}{\underset{︸}{\sum_{n}B_{n}\cos\left(2\pi nTf_{0}t+\beta_{n}\right)}}\\ +\underset{Defectimpulses}{\underset{︸}{\sum_{i}D_{i}S_{d}\left(t-iT_{d}-\tau_{i}\right)}}+\underset{Randomshocks}{\underset{︸}{\sum_{i}R_{i}S_{r}\left(t-T_{i}\right)}}+n\left(t\right) \end{array}$$

The first part is the vibration from the rotor or shaft, where *f*_0_ is the rotation frequency, *A_n_* and *a_n_* represent the amplitude and phase of the *n*th harmonic, respectively. The vibration signal originating from gear meshing is represented by the second part, where the number of gear teeth is denoted as *T*, and the amplitude and phase of the *n*th meshing frequency are represented by *B_n_* and *β_n_*. The third part represents the defect impulses caused by the failure of the rolling bearing, where the amplitude of the *i*th impulse is represented by *D_i_,* and *T_d_* represents the interval time between the two adjacent impulses. Moreover, *τ_i_* is used to simulate the random slip of the bearing, which is usually set to 1~2% of the fault period.

In general, the defect impulses can be represented as an exponentially decaying sinusoid, as follows:

(16) $$S_{d}(t)=e^{\left(-\alpha_{r}t\right)}sin(2f_{r}t)$$

where *f_r_* is the resonant frequency of the impulse response and *α_r_* is the damping ratio of the impulses.

The fourth part is the external random shock, where *R_i_* and *T_i_* denote the amplitude and occurrence time of the *i*th random shock, respectively. Furthermore, *n*(*t*) represents white Gaussian noise.

**Figure 4:**
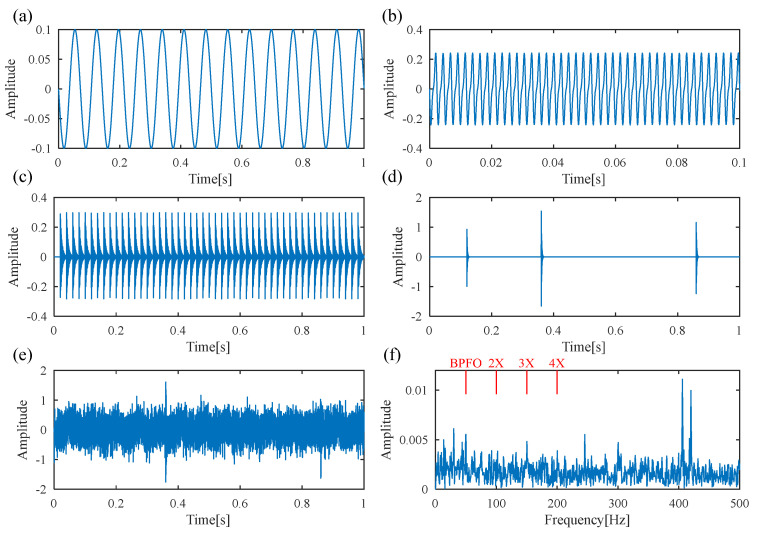
Simulated signal. (**a**) rotor vibration; (**b**) gear meshing; (**c**) defect impulses; (**d**) random shocks; (**e**) noise-added mixture signal; (**f**) envelope spectrum.

Table 1 lists the parameters of the simulated signal. In the simulation, the time length of the simulated signal and sampling frequency are set as 1 s and 10,000 Hz, respectively. Figure 4a–d display the time waveforms of the rotor vibration, gear meshing, defective impulses and random shocks, respectively. After adding white Gaussian noise with an SNR of −3 dB, the synthetic signal is shown in Figure 4e, and no obvious defect impulses can be recognized. The envelope spectrum of the simulated signal is presented in Figure 4f, where the frequency lines of the BPFO are ambiguous, and no signature indicates the existence of a bearing fault. Here, the BPFO represents the FCF of the outer race.

**Figure 5:**
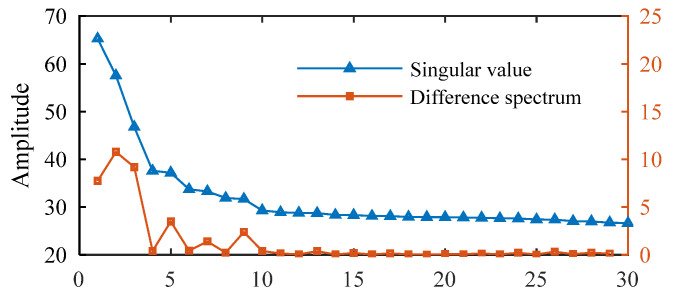
Singular values and difference spectra.

**Figure 6:**
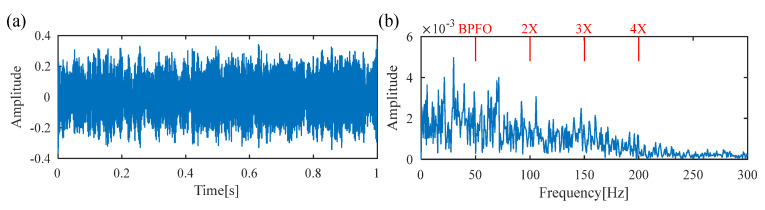
The results obtained by DS-SVD for simulated signal. (**a**) denoized signal; (**b**) envelope spectrum.

**Figure 7:**
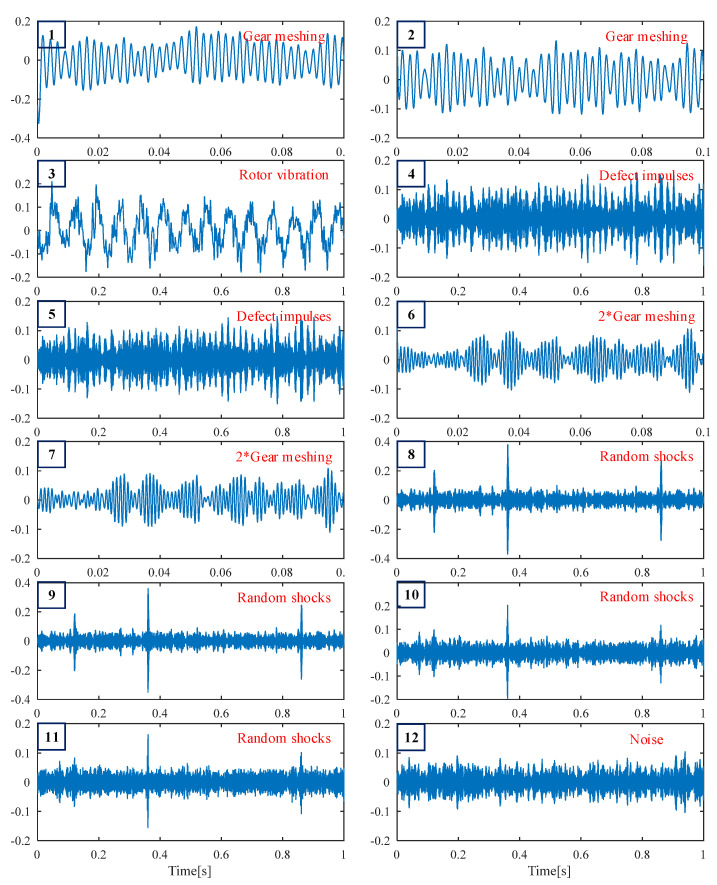
The first 12 singular components obtained using SVD.

Next, the traditional DS-SVD [25] and the proposed FDMK-SVD are applied to analyze the simulated vibration signal. In traditional DS-SVD, the threshold *k* is determined by the difference spectrum (DS), and the fault information is considered to be contained in the first *k* SCs. Figure 5 and Figure 6 show the results obtained using DS-SVD. According to the difference spectrum shown in Figure 5, the maximum peak is observed at *k* = 2, which means that only the first two singular components are selected to reconstruct the denoized signal. Figure 6a presents the denoized signal, and no obvious defect impulses are observed. The envelope spectrum of the reconstructed signal is displayed in Figure 6b, and it also fails to provide useful information for fault diagnosis. To further investigate the reason for this phenomenon, the decomposition results obtained using SVD are shown in Figure 7. As shown in Figure 7, the first two SCs selected for reconstructing the denoized signal based on DS-SVD are composed of the harmonic components originating from gear meshing, and the defect impulses are ignored in the DS-SVD. The above results indicate that the selection scheme of traditional DS-SVD is inappropriate when the diagnostic information is very weak and disturbed by other large-energy components.

**Figure 8:**
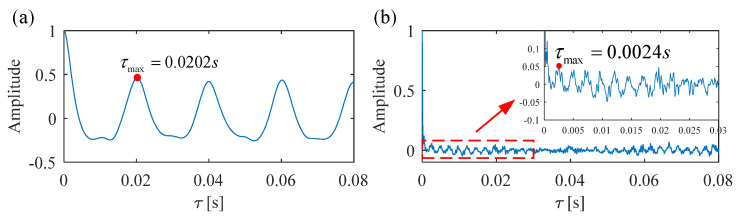
Envelope autocorrelation function of (**a**) selected singular component and (**b**) raw signal.

**Figure 9:**
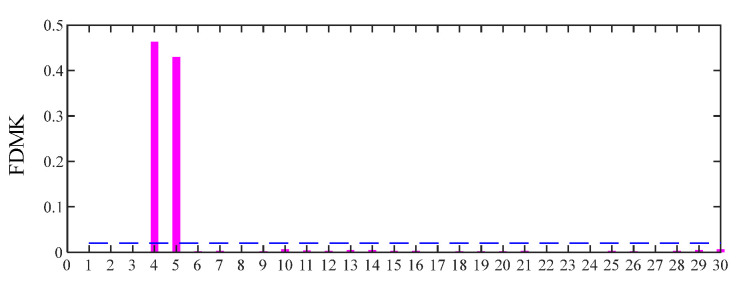
FDMK of each singular component for simulated signal.

**Figure 10:**
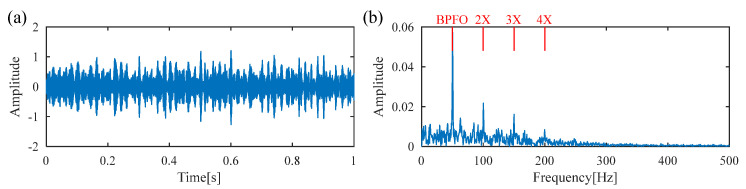
The results obtained by FDMK-SVD for simulated signal. (**a**) denoized signal; (**b**) envelope spectrum.

For comparison, the same simulated signal is processed using the proposed FDMK-SVD. The envelope autocorrelation function of the selected most sensitive SC and raw signal is displayed in Figure 8. As shown in Figure 8a, the local maximum is located at *τ*_max_ = 0.0202 s, and its corresponding frequency is 49.505 Hz, which has a slight deviation from the real value. On the contrary, the fault period is masked by the harmonic period (*τ*_max_ = 0.0024 s) corresponding to gear meshing when the raw signal is used to estimate the period or FCF. The reason for this phenomenon is that the energy of the defect impulses is less than that of the harmonic components. After obtaining the estimated fault characteristic frequency, the target vector $\vec{t}$ is constructed, and the FDMK value of SC is calculated and is displayed in Figure 9. As shown in Figure 9, although the first three SCs have large energies, their FDMKs are very small since they are not relevant to the bearing fault. On the contrary, the FDMK of the 4th and 5th components are significantly larger than those of the others. By checking the decomposition results obtained by SVD shown in Figure 7, the 4th and 5th SCs correspond to the defect impulses, which is significant information for identifying the health conditions of bearings. The time waveforms of the denoized signal and the corresponding envelope spectrum are displayed in Figure 10. According to Figure 10, the defect impulses are successfully extracted in the time domain, and BPFO and its harmonics are clearly recognized in the envelope spectrum. There is no doubt that the fault is successfully detected by the proposed FDMK-SVD.

To evaluate the computational efficiency of the proposed method, the execution time is analyzed on a laptop equipped with a 12th Gen Intel^®^ Core™ i7-12700H processor (2.7 GHz) and 16 GB DDR4 RAM, running MATLAB R2022a. The execution time for analyzing the simulated signal was 1.563 s. This relatively short processing time highlights the proposed method’s suitability for real-time applications, including online monitoring. Therefore, the proposed method not only provides accurate fault diagnosis but also ensures computational efficiency, making it ideal for condition monitoring of bearings.

## 5. Experimental Verification

**Figure 11:**
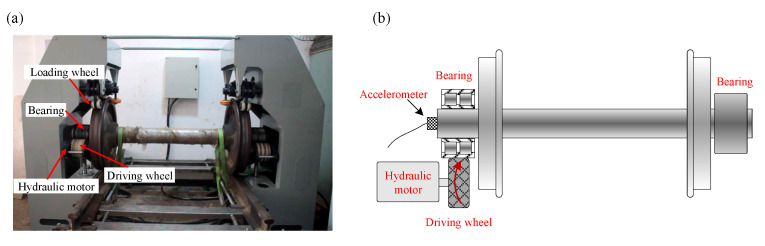
(**a**) Experiment setup of the test rig; (**b**) schematic view of the locomotive bearing test rig.

In this section, an experimental test is conducted on a locomotive bearing test rig to verify the validity of the proposed FDMK-SVD. The test rig consists of a driving wheel, a hydraulic motor, and a locomotive wheel, as shown in Figure 11. Notably, in this setup, the outer race of the bearing is driven by the hydraulic motor, while the inner race remains fixed. As a result, when a fault occurs in the outer race, amplitude modulation occurs, and sidebands are expected to appear in the envelope spectrum. In contrast, no amplitude modulation is observed for faults in the inner race. The vibration signal is collected by a three-axis PCB accelerometer with a sensitivity of 100 mV/g, which is placed at the end of the shaft. The sampling frequency is set to 76,800 Hz, and the data length is 1 s. Moreover, the speed information of the driving wheel is also acquired by a tachometer. In the experiment, the bearing under test is a tapered roller bearing, model 197,726. The geometric parameters of the locomotive bearings are listed in Table 2. To simulate real-world scenarios, the test rig was subjected to various noise sources, including mechanical vibrations from the surrounding machinery and environmental noise.

### 5.1. Experiment 1: The Bearing with Inner-Race-Fault

In this case, in order to further validate the validity of the proposed FDMK-SVD, the vibration signal from a bearing with an inner-race fault is processed. Table 3 lists the FCFs of the tested inner-race-fault bearing, where *f_r_* specifies the rotating frequency of the outer race, and BPFO, BPFI, and BSF represent the FCF of the outer race, inner race, and rolling element, respectively.

**Figure 12:**
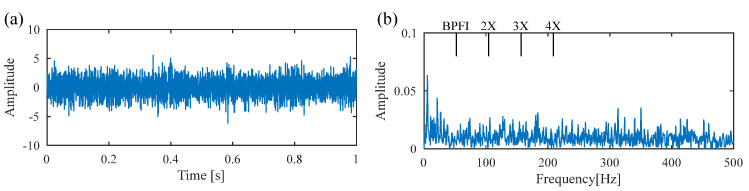
(**a**) Raw signal of the inner race fault bearing; (**b**) envelope spectrum.

**Figure 13:**
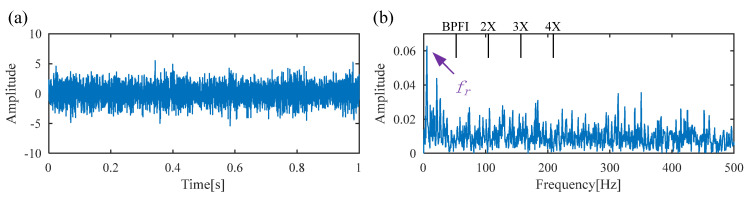
The results obtained by DS-SVD for the signal of the inner race fault bearing. (**a**) denoized signal; (**b**) envelope spectrum.

Figure 11 displays the raw vibration signal and its envelope spectrum. Owing to the heavy noise, the periodic defect impulses cannot be obviously observed from the time waveform, as shown in Figure 12a. Meanwhile, no significant BPFI or its harmonics are observed in the envelope spectrum, as shown in Figure 12b. To extract the weak fault feature from the raw vibration signal, DS-SVD is applied, and the corresponding denoized signal is illustrated in Figure 13. However, useful information related to the inner-race fault cannot be observed from the waveform of the denoized signal or its envelope spectrum. Moreover, it can be seen that the rotating frequency *f_r_* is dominant in the envelope spectrum, which illustrates that the DS-SVD method, which relies on an energy-oriented selection scheme, fails to effectively select the sensitive SCs containing diagnostic information when harmonic components dominate the signal.

**Figure 14:**
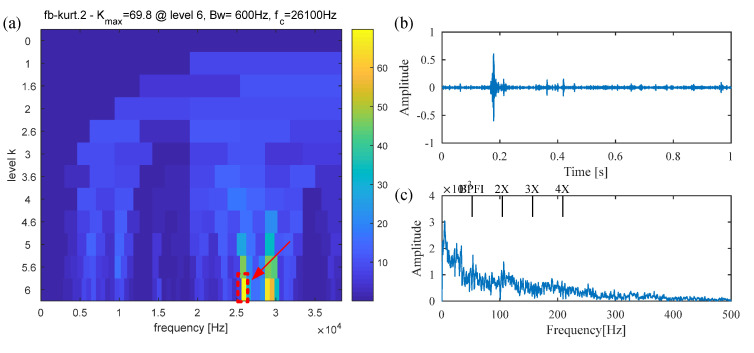
The results obtained by the SK method for the signal of the inner race fault bearing. (**a**) Kurtogram; (**b**) filtered signal; (**c**) envelope spectrum.

Spectral kurtosis (SK), a widely used and state-of-the-art method, is also adopted to extract the fault feature from the raw signal. Figure 14a presents the Kurtogram of the raw vibration signal, from which the resonance band with maximum spectral kurtosis (center frequency: 21,600 Hz; Bandwidth: 600 Hz) is easily determined. A band-pass filter with a center frequency of 21,600 Hz and a bandwidth of 600 Hz is designed to filter the vibration signal, and the filtered signal is displayed in Figure 14b. However, only several random impulses can be observed, and the periodic fault impulses are not detected. Similarly, the envelope spectrum in Figure 14c shows no obvious fault information. This failure results from the assumption that fault impulses occur in the frequency band with the largest kurtosis value. However, kurtosis is only a statistical indicator of signal sparsity, and the periodicity of the defect impulses is not taken into consideration. Therefore, it is susceptible to interference from non-Gaussian signals and random noise.

**Figure 15:**
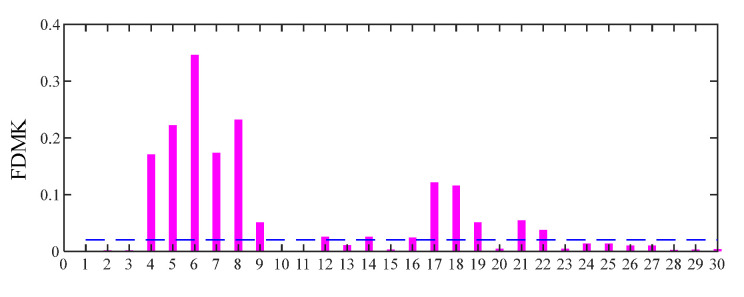
FDMK of each singular component for the signal of the inner race fault bearing.

**Figure 16:**
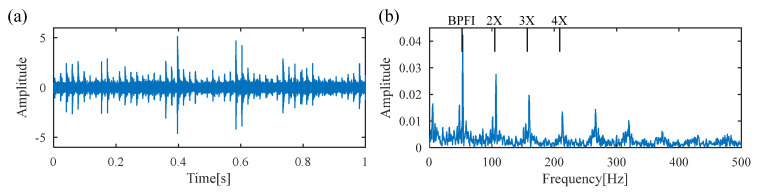
The results obtained by FDMK-SVD for the signal of the inner race fault bearing. (**a**) denoized signal; (**b**) envelope spectrum.

**Figure 17:**
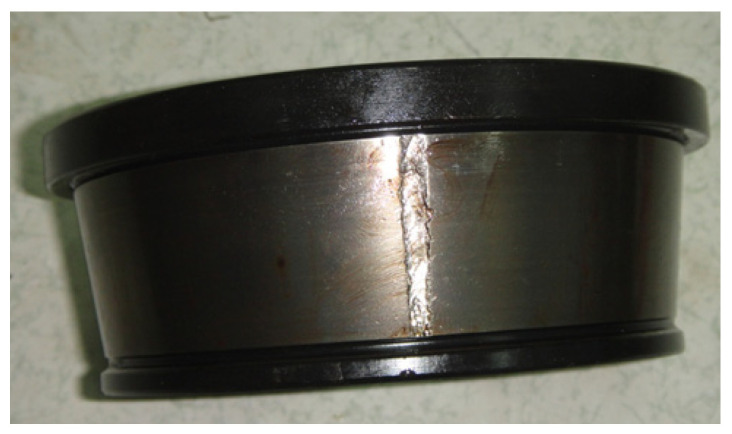
The fault on the inner race.

For comparison, the proposed FDMK-SVD is conducted on the same vibration signal. The FDMK value of each SC is calculated and is displayed in Figure 15. The denoized signal and its envelope spectrum are displayed in Figure 16. As expected, defect impulses are effectively detected, which illustrates the effectiveness of FDMK-SVD in extracting fault features and suppressing noise interference. Moreover, the BPFI and its harmonics are clearly observed, which provides evidence of the presence of an inner-race fault. To verify the results of the above analysis, the test bearing is disassembled, and the picture is illustrated in Figure 17, from which the inner-race fault of the bearing is clearly identified.

### 5.2. Experiment 2: The Bearing with Outer-Race Fault

In this case, the vibration signal from a bearing with an outer-race fault is used to further verify the effectiveness of FDMK-SVD in detecting bearing faults. Table 4 lists the fault characteristic frequencies for the outer-race fault.

**Figure 18:**
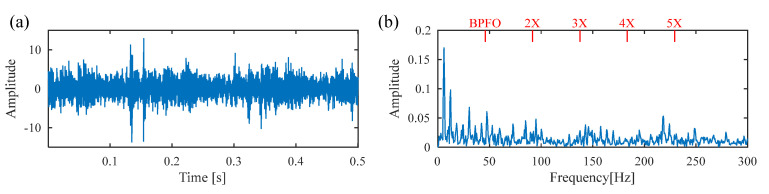
(**a**) Raw signal of the outer race fault bearing; (**b**) envelope spectrum.

**Figure 19:**
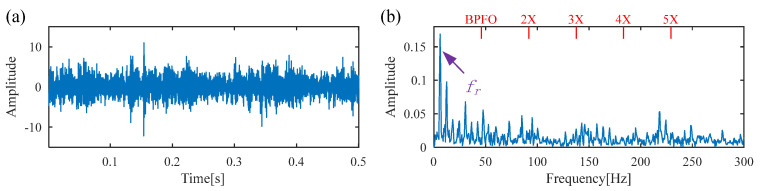
The results obtained by DS-SVD for the signal of the outer race fault bearing. (**a**) denoized signal; (**b**) envelope spectrum.

**Figure 20:**
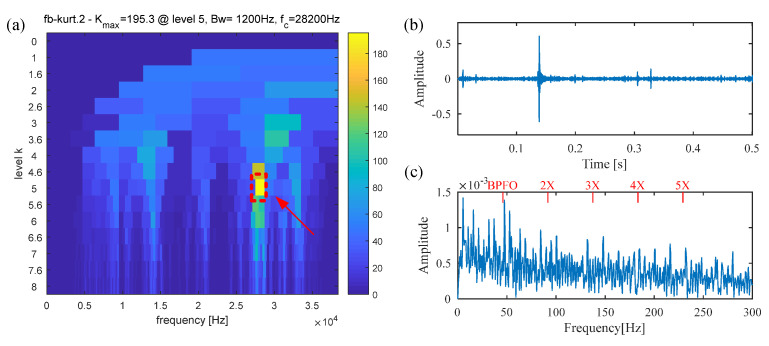
The results obtained by the SK method for the signal of the outer race fault bearing. (**a**) Kurtogram; (**b**) filtered signal; (**c**) envelope spectrum.

**Figure 21:**
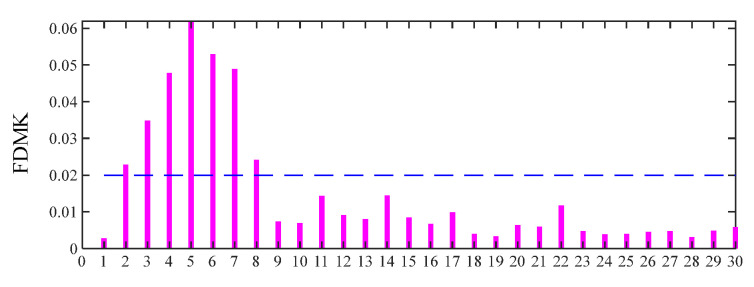
FDMK of each singular component for the signal of the outer race fault bearing.

**Figure 22:**
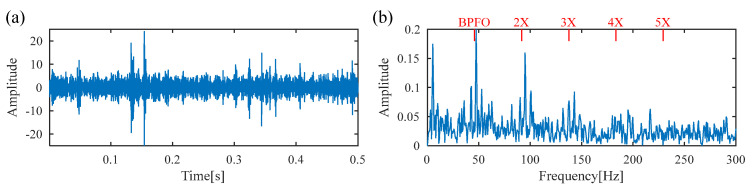
The results obtained by FDMK-SVD for the signal of the outer race fault bearing. (**a**) denoized signal; (**b**) envelope spectrum.

**Figure 23:**
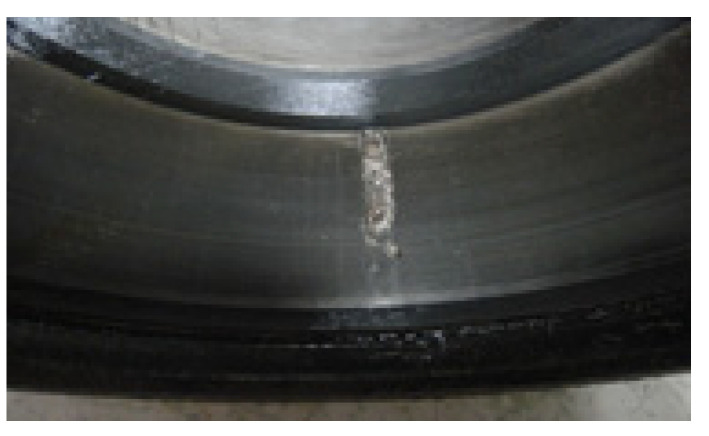
The fault on the outer race of the bearing.

Figure 18 presents the time waveform of the raw vibration signal and its envelope spectrum, from which no periodic fault impulses or fault characteristic frequency can be observed. Then the DS-SVD is performed on the vibration signal, and the results are displayed in Figure 19. Similar to Experiment 1, it can be seen that the rotating frequency and its multipliers are dominant in the envelope spectrum, while the BPFO and its harmonics are not prominent, which implies that the suitable SCs with abundant information are not identified. Figure 20a shows the Kurtogram of the signal, where the resonance band is determined to be at the center frequency of 282,000 Hz with a bandwidth of 1200 Hz. The filtered signal obtained through the corresponding band-pass filter is presented in Figure 20b, where only random shocks are extracted. Moreover, there is no obvious fault characteristic frequency or its multiplier in the envelope spectrum. Finally, the proposed FDMK-SVD is applied to the same signal, and the results are shown in Figure 21 and Figure 22. According to Figure 21, it can be seen that the FDMK of several SCs exceeds the threshold line, which implies that these SCs may contain useful diagnostic information. To validate the above analysis, a reweighting scheme is adopted, and the reconstructed signal is shown in Figure 22. Periodic fault impulses are effectively extracted, which verifies the effectiveness of FDMK-SVD in extracting weak fault features and suppressing noise interference. Moreover, the BPFO and its harmonics are obvious in the envelope spectrum, which indicates that the outer race of the rolling bearing is faulty. Then the locomotive bearing is disassembled, from which the outer-race fault of the bearing is clearly recognized, and the picture is illustrated in Figure 23.

## 6. Conclusions

This paper proposes a novel method, FDMK-SVD, for the diagnosis of rolling element bearings. In this work, the vibration signal is firstly decomposed via SVD, and then the sensitive SCs containing diagnostic information are selected and reweighted based on the FDMK index to reconstruct the denoized signal. Meanwhile, the estimation process of fault characteristic frequency is incorporated into the proposed method to address the issue that prior information about FCF is not predetermined in practice. Using the proposed method, weak fault features indicating the occurrence of bearings are successfully extracted. Moreover, the effectiveness and advantages of FDMK-SVD in detecting bearing faults are validated by comparing it with DS-SVD and Kurtogram. The results demonstrate that FDMK-SVD is a promising method for the condition monitoring of bearings, even in the presence of heavy noise and strong random interference.

The proposed FDMK-SVD has been validated for a constant-speed operation; however, real-world machinery often operates at varying speeds. Future work will focus on adapting SVD-based signal analysis to variable-speed conditions.

## Figures and Tables

**Table 1:** The parameters of the simulated signal.

Rotor and Shaft			Gear Meshing					Defect Impulses				Random Shocks	
*f*_0_	*A*_1_	*α*_1_	*T*	*B*_1_	*β*_1_	*B*_2_	*β*_2_	*D*_1_	*T_d_*	*f_r_*	*α_r_*	*f_r_*	*α_r_*
14	0.1	*π*/2	30	0.2	*π*/2	0.08	*π*/2	0.3	1/50	2400	200	4000	700

**Table 2:** Geometric parameters of the locomotive bearing.

Number of Rollers	Pitch Diameter (mm)	Roller Diameter (mm)	Contact Angle (Degree)
20	180	23.775	9

**Table 3:** Bearing characteristic frequencies (Hz) in Experiment 1.

***f***_***r***_	BPFO	BPFI	BSF
4.6183	40.1584	52.2083	17.1851

**Table 4:** Bearing characteristic frequencies (Hz) in Experiment 2.

***f***_***r***_	BPFO	BPFI	BSF
5.2747	45.8657	59.6281	19.6274

## Data Availability

Data are unavailable due to privacy restrictions.
